# GIS-aided optimisation of faecal sludge management in developing countries: the case of the Greater Accra Metropolitan Area, Ghana

**DOI:** 10.1016/j.heliyon.2019.e02505

**Published:** 2019-09-21

**Authors:** Gideon Sagoe, Felix Safo Danquah, Eric Simon Amofa-Sarkodie, Eugene Appiah-Effah, Elsie Ekumah, Emmanuel Kwaw Mensah, Kenneth Sefa Karikari

**Affiliations:** a Sewerage Systems Ghana Ltd, P. O. Box GP 1630, Accra, Ghana; b Department of Civil Engineering, Kwame Nkrumah University of Science and Technology, Kumasi, Ghana

**Keywords:** Chemical engineering, Environmental engineering, Environmental management, Environmental pollution, Geography, Sanitation, Faecal sludge, Spatial analysis, GIS, Onsite sanitation system, Lavender Hill FTP, Greater Accra Metropolitan Area

## Abstract

This study employed GIS tools to help optimise faecal sludge (FS) management in the Greater Accra Metropolitan Area (GAMA) and its environs in Ghana. First, the rates of excreta generation, FS generation and FS collection were quantified based on literature, census and FS discharge data obtained from treatment plants in the study area. Next, we mapped the FS collection to the administrative areas in GAMA based on discharge records obtained from Lavender Hill, the main faecal treatment (FTP) and estimated the travel distance and travel time from the various FS desludging neighbourhoods to the plant. The results of the study show that the excreta and FS generation rates in GAMA are 604 L/cap/yr and 4,137 L/cap/yr, respectively. About 1 million m^3^ of FS was collected and treated in the study area in 2018, with a collection rate of 244 L/cap/yr. The private sector dominates this collection, haulage and treatment of FS in GAMA. The GIS analysis has provided fundamental data that will be useful in rationalising the FS emptying and transport cost in the study area. Moreover, it revealed that about 20–40% of the localities were outside the 15–25 km sustainable maximum transport distance recommended by some scholars. Finally, the findings highlight the importance of looking beyond administrative boundaries when planning for FS management logistics and infrastructure and also show that the most impoverished communities in the Accra metropolis may not necessarily be the least served when it comes to FS collection and haulage.

## 1 Introduction

### 1.1 Background

Globally, sanitation is a topical issue due to its close connection with human health and dignity and the environment. However, there is still a gap in providing proper sanitation in developing countries. For example, in Africa, over 60% of the population does not have access to improved sanitation, while 40% of the rural populace resort to open defecation [1]. Targeted initiatives ensuring access to safe water and sanitation for all by the year 2030, as stated by goal six of the Sustainable Development Goals (SDGs), are of emergent implementation for many developing countries, especially those in sub-Saharan Africa and South Asia [2].

Sanitation can be managed by centralised, semi-centralised or on-site sanitation systems. For a centralised sanitation system, wastewater (composed of faecal matter, urine and greywater) is transported via sewer lines from a large catchment area to a treatment plant. A semi-centralised sanitation system is usually used to serve neighbourhoods or a cluster of homes and institutions via relatively short sewer systems. Onsite sanitation systems (OSSs) are used to treat excreta and wastewater, either partially or fully, at the point of generation [3]. The choice of sanitation management option implemented chiefly depends on factors such as available resources, population, the socio-economic disposition, the legal and institutional conditions, and the general development planning concept of an area [4]. Most middle to low-income countries are dominated by OSSs [5, 6] because they serve as a more economically sustainable option [7].

In Ghana, only 4.5% of the country's population is connected to sewer networks [8]. In Accra, the capital city, OSSs such as septic systems, pit latrines, and ventilated improved pits are the most common. This is because only about 15% of the total land area of the central business district, the Accra Metropolis, is connected to a sewer network [9]. In OSSs, faecal sludge (FS) accumulates over time, requiring periodic emptying of the tanks [10]. FS refers to either the raw or partially digested slurry or semi-solid generated form of excreta in the septic tanks and pit latrines and can take few weeks to several years before it is ready to be removed [3, 11].

### 1.2 Faecal sludge management in Accra

The faecal sludge management (FSM) value chain includes collection (emptying), haulage, treatment, and safe reuse or disposal of FS. Effective FSM requires that all the aspects of the chain are well-managed in a sustainable manner [12]. In Accra, FS collection and transport are mainly done by vacuum tankers [12, 13, 14]. However, manual collection is still done on a relatively small scale [14]. In the year 2006, about 200,000 m^3^ of FS was collected and dumped into the Atlantic Ocean without treatment [12]. In 2010 this figure increased to about 550,000 m^3^, as inferred from Koppelaar et al. [13].

Over the years, Accra has had three major FS treatment plants, located at Achimota, Teshie-Nungua and Korle Gonno (Old Lavender Hill) [15, 16, 17], but Achimota and Korle Gonno have since been decommissioned. The Teshie-Nungua waste stabilisation ponds (10,000 metric tonnes capacity) [27] receive a daily FS loading of 80–100 m^3^ [9], [16]. Currently, Accra has several operational FS treatment plants with relatively modern technologies being used, the most prominent of them being the 2,000 m^3^/day capacity Lavender Hill faecal treatment plant (FTP). In addition to this, there is the Slamson Ghana cesspit treatment plant (400 m^3^/day capacity) situated at the Old Lavender Hill and the Kotoku FTP (1,000 m^3^/day capacity). The Safi Sana waste-to-energy plant (WTEP) and the Jekora Ventures Limited Fortifier Compost Plant (FCP) also use FS for the production of organic fertilisers and biofuel.

Despite the availability of these treatment facilities, the high cost of FS collection and haulage (C&H) in Accra and its environs is a challenge to FSM. Boot and Scott [12] reported that the increase in the cost of FS C&H in the past decade was due to the cost of long hauling distances. Specifically, this increase in cost was due to the closure of Achimota WSP, formerly located in the northern part of Accra, as this caused drivers to haul the FS longer distances than usual.

### 1.3 Quantification of faecal sludge

Efficient management of FS requires knowledge of the quantitative and qualitative characteristics of the FS generated and the options available for handling FS safely for beneficial use. However, estimating FS generation on a city-wide scale is complicated due to several factors. For a reasonable estimate, information on the number of users, location, types and number of OSS, FS accumulation rate and the socioeconomic levels of the population are required. Moreover, there are no proven methods for accurately quantifying FS, as the collection of the data required is difficult [18]. However, Strande et al. [19] recently presented a more systematic and reliable approach, which breaks down FS quantification into six phases. The six phases stated are: (1) excreta generated, (2) FS produced, (3) FS accumulated in the OSSs, (4) FS emptied from OSSs but not collected, (5) FS collected but dumped into the environment, and (6) FS collected and delivered for treatment. In this study, surveys and data recorded at an FTP were used to quantify FS collected in GAMA.

### 1.4 GIS-aided optimisation of faecal sludge management

Geographic information system (GIS) offers useful tools to spatially analyse faecal sludge facilities and logistics for FS management optimisation. It increases how sustainable the planning and decision-making processes are, the accessibility of services, and also reduces the cost and transportation times of FS [20]. In Switzerland, GIS tools were used to identify the suitability of siting decentralised and centralised systems in regions of different population density, among others [21]. As part of the EPA Research Program in Ireland, GIS analysis helped to reveal the gaps in infrastructural and FS transport and processing requirements for the whole country [22]. In Uganda, similar approaches were adopted to analyse the service coverage of cesspit emptying service providers, the proximity of FS sources to the locations of existing and proposed treatment plants and the influence of population density on FS discharge frequency in the city of Kampala [20]. Such approaches, however, remain untapped for FSM in Ghana.

Furthermore, knowledge of the distance from sanitary installations to the treatment plant is useful in calculating the haulage cost per cubic meter of faecal sludge collected and transported, using equations provided in the literature [5, 16]. Two key parameters for the computations are the travel distance and travel time. By the use of GIS, such data (travel distance and time) can be obtained to help in optimising FS management in Ghana, especially in the determination of reasonable service charges by the vacuum tanker operators. Since equitable access to sanitation transcends the provision of toilet facilities [20], ensuring fair C&H service charges is one of the ways towards achieving Goal 6 of the SDGs [23]. Apart from FS C&H cost rationalisation, proper siting of FTPs can help reduce fuel consumption, thereby decreasing the GHG emissions by a country, contributing to the global sustainability agenda.

Therefore, the aim of this study was to employ GIS tools to help optimise FS management in GAMA and its environs. The specific objectives were to (1) estimate the quantities of FS (excreta generation, FS generation, FS collected and FS treated) in the study area, based on the methods provided by Strande et al. [19]; (2) map the collected FS discharged at the Lavender Hill FTP to the various neighbourhoods and Metropolitan/Municipal/District Assemblies (MMDAs) in GAMA and its environs; (3) estimate the average travel distances and minimum travel times from the various FS sources to the treatment plant, to provide fundamental data for rationalising FS collection and haulage cost in the study area; (4) and finally, assess the influence of population density and income levels of residents in the neighbourhood level on the level of service coverage by the tanker operators in GAMA. The estimated FS quantities will allow comparison with previous data to assess if GAMA is making progress proper FSM and also compare the city's performance with other cities. The GIS analysis will also provide information on the service coverage of the Lavender Hill FTP and the FS loadings from the MMDAs treated by the facility. Also, the FS quantities and spatial analysis will be useful to city authorities in siting new treatment plants in the study area.

## 2 Materials and methods

### 2.1 Study area

**Fig. 1:**
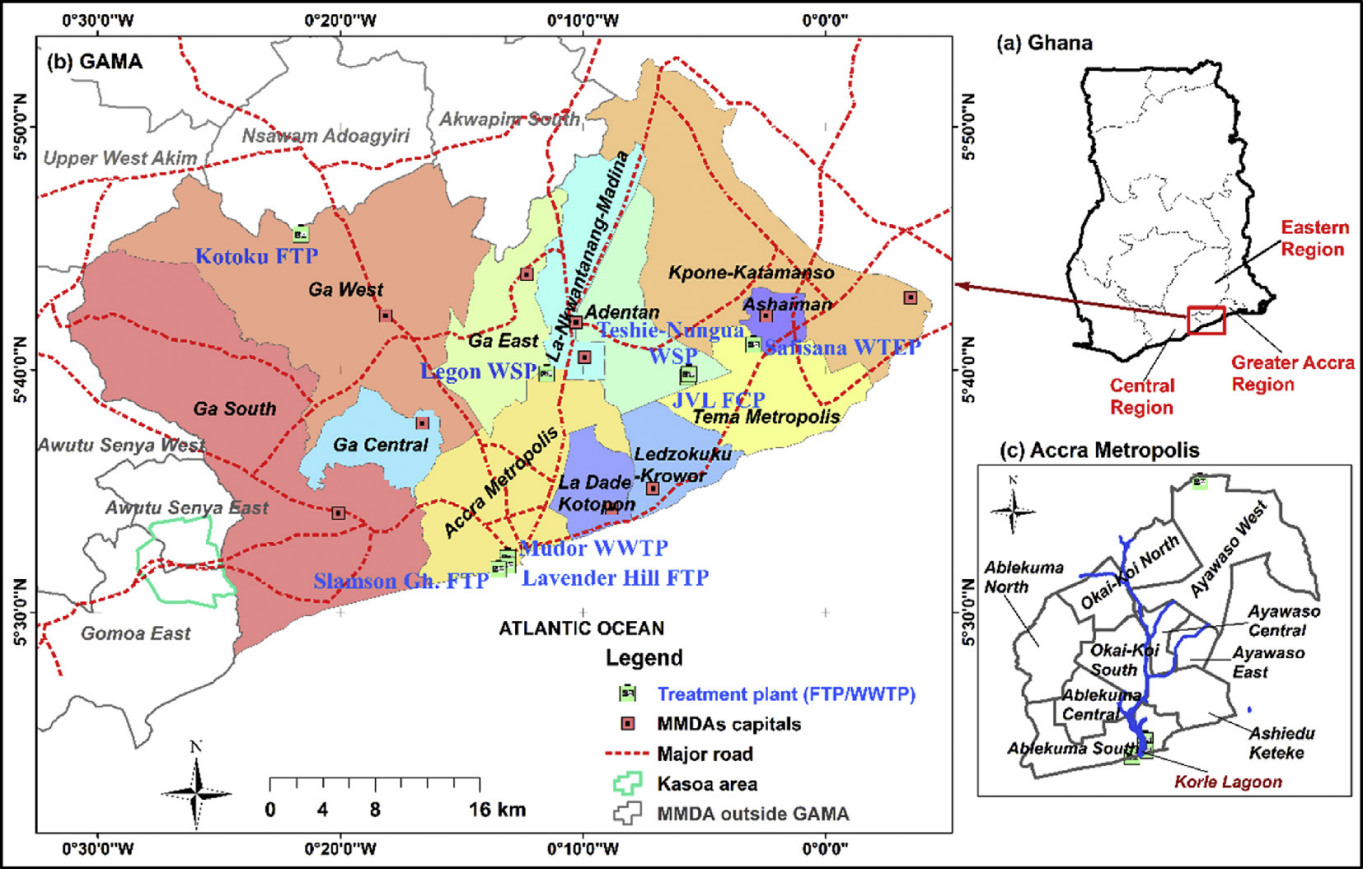
Map of the study area.

The study focused on GAMA, which is located in the Greater Accra region of Ghana, and Kasoa, a peri-urban community in the Central Region (see Fig. 1). Over the years, GAMA has undergone several changes in the number of administrative areas and boundary demarcations [13]. However, this study used the boundaries based on the 2010 Population and Housing Census [24, 25, 26, 27, 28, 29, 30, 31, 32]. GAMA covers about 1,500 km^2^ and consists of the Accra Metropolis (AMA), the Ga municipalities (Ga West, Ga South, Ga Central and Ga East), the Tema Metropolis, the Ashaiman Municipality and Kpone Katamanso district, collectively referred to as the GAMA MMDAs (Metropolitan, Municipalities and District Assemblies). The total population in GAMA is currently about 4.2 million [33]. Accra, the capital city of Ghana, is located in GAMA. The AMA is sub-divided into eight sub-metropolitan districts, as shown in Fig. 1. As a central business district in GAMA, the AMA has many slum communities [34].

This study also featured the Kasoa area because, from our experience at the Lavender Hill FTP, it contributes to a significant volume of FS daily received at the plant. Kasoa has land coverage of about 35 km^2^ and about 69,300 inhabitants [35]; it is located at the western periphery of the Greater Accra region. Kasoa is one of the ‘spill-over’ areas that receive a high number of migrants in Accra [35, 36]. The town's population growth rate is 3.3% [37], which is about two times the average of the Central region (1.8%) [38]. The climatic conditions (rainfall and temperature) in Kasoa are similar to those in Accra.

### 2.2 Data collection

#### 2.2.1 Questionnaire survey

A semi-structured questionnaire was designed and administered to the vacuum tanker drivers to gain some information on the collected FS. We used a semi-structured interview because it provides a flexible and open two-way communication along a topical trajectory [39]. Since the number of trucks that discharged at the plant was not known prior to the study, we assumed a population of 70 truck drivers based on the average number the drivers who attended meetings with the managers of the Lavender Hill facility. A sample size of 60 was calculated (at 95% confidence level and 5% margin of error) as the number of targeted participants. The participants were randomly sampled whiles they discharged at the plant, over the course of a week; each interview took an average of about 4 minutes. The questions sought to obtain information on the capacity of truck; ownership of truck (private or government); whether the vacuum tanker is fully or partially filled before discharging at the treatment plant; how many OSSs are emptied in a trip; whether the customer's onsite FS containment tank is fully emptied; whether water is added to the containment system before an emptying episode; the drivers' preference between the Lavender Hill FTP and the Kotoku FTP, with reason(s); and lastly, whether they sometimes dump collected FS directly into environment.

#### 2.2.2 Direct measurement of truck capacity

To ensure the reliability of the estimated truck volumes deduced from Section 2.2.1, we conducted field measurements (length, L and diameter, D) of the tanks of 100 trucks over another week. The volumes of the tanks were estimated by the formula ^1^/_4_ πD^2^ L, with the assumption that the tanks were perfect cylinders. In cases where the vacuum tankers had both water storage and FS haulage compartments, only the portion of the tanks for conveying FS was measured.

#### 2.2.3 Administrative boundary settings and population data

We collected the most current census data (2010 Population and Housing Census) on the usage of sanitation facilities and methods liquid and solid waste disposal in the districts [24, 25, 26, 27, 28, 29, 30, 31, 32, 35]. The 2018 projected neighbourhood-scale data for the localities in the Accra Metropolis was obtained from the Planning Division of the Accra Metropolitan Assembly. Data on the socioeconomic levels of neighbourhoods in the AMA was obtained from a 2013 report by the UN-HABITAT [34].

#### 2.2.4 Spatial analysis faecal sludge collection and discharge data

**Fig. 2:**
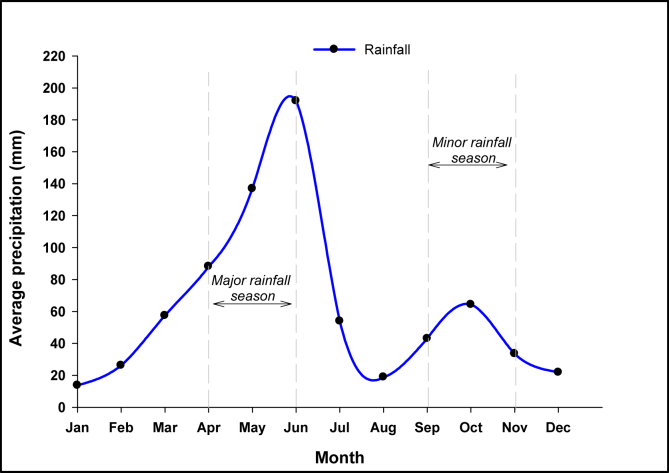
Rainfall hydrograph of Accra based on data from the year 1951–2010 [41].

This analysis adopted the sludge collection method [18] to quantify FS in the study area. The research focussed on FS emptying events at the Lavender Hill FTP, since it is a sink for about 60% of the FS collected in the study area (see Table 2 in Section 3.3). Since the FS discharge frequency at some dumping sites and treatment plants has been associated with rainfall pattern [18, 20, 40], we first analysed available precipitation data [41] to identify the rainfall patterns, and subsequently used that to select a suitable period for our study. As shown in Fig. 2, Accra has two rainy seasons. The major wet season is from April to the middle of July and the minor wet season, from September to November. Therefore, all available information on the sources of FS received at the Lavender Hill FTP from 1^st^ January to 30^th^ June 2018 was collated to capture one dry and the major rainy season. The information on the FS source is provided by the truck drivers for every trip and recorded. However, there were missing source data for some of the recorded events (from 1^st^ to 3^rd^ January 2018). Therefore, in the calculation of neighbourhood-sensitive parameters, such as the FS generation rate and the discharge frequency of an area, we excluded any discharge event with missing FS source information. Occasionally, industrial wastes (dairy products and pharmaceutical wastes) are discharged at the plant, so all non-faecal sludge emptying events were excluded. We then identified all the well-known neighbourhoods (FS sources) recorded over the study period; the unpopular communities were added to the nearest famous towns, provided they were in the same MMDA.

Since the Lavender Hill FTP is not the only sink for the collected FS in GAMA, the total number of discharge events registered at the Kotoku FTP, Slamson FTP, Safi Sana WTEP and the Jekora FCP were collected. However, this data was utilised in quantifying the total FS collected and discharged at treatment plants.

The ESRI shapefiles for the demarcation the administrative boundaries and towns were obtained from online (http://www.rsgislab.ug.edu.gh/content/data-sharing-app), and that of the major road network from the Mapcruzin website (https://mapcruzin.com/ghana-shapefiles/ghana_highway.zip). There were no available shapefiles for the boundaries of the sub-metropolises and neighbourhoods in the Accra Metropolis. Thus, we digitised geo-referenced maps from [42] and [34] to define the boundaries for the two levels, respectively.

To determine the travel distance and time from an FS source to a treatment plant, heuristic algorithms are usually needed to first identify the route(s) from the FS source to the discharge point. The calculations of the time and the distance between the points of interest take into account factors such as the characteristics of the geometric space [21]. The study area has a relatively good road networking system because of its rapid urbanisation; this has made possible the operation of commercial online transportation platforms in the city. Hence, road network analysis was considered to be reliable for this study. We employed the heuristic routing algorithms of Google Earth Pro (version 7.3.2.5491) to estimate the average minimum distance travelled and the minimum time taken by a vacuum truck from the source (locality) to the treatment facility. For each FS source, all the possible routes to the treatment plant were included in the calculations. For each MMDA or sub-metropolis, the average distance and the haulage time were calculated based on all the FS sources identified within its boundaries. All collected data were integrated into an MS Excel spreadsheet for analysis.

### 2.3 Data analyses

#### 2.3.1 Faecal sludge quantification

Based on the 2010 population data and FS collection data obtained at the Lavender Hill FTP and other plants which act as collected FS sinks, we quantified FS in GAMA at different stages, as described by Strande and others [19]. The stages included excreta generation rate (Q_1_), FS generation rate (Q_2_), FS emptied but not collected, FS collected but discharged into the environment (Q_3_), and FS collected and treated (Q_4_). Q_1_ was estimated by Eq. (1), and Q_2_ by Eq. (2), Eq. 3 and Eq. 4 [19]. Table 1 shows the estimated FS quantities in GAMA. For the Q_2_ estimate, the proportions of the population that are connected to the sewer networks in the Accra metropolis (7.8%) [24] and Tema Municipality (39.9%) [30] were excluded. Also, the volume of solid waste disposed of in the containment systems was factored in the calculations [18].

(1) $$Excreta\text{ ​}production\text{ ​}(Q_{1})=P_{\left(served\right)}\times (Q_{\left(urine\right)}+Q_{\left(faeces\right)})$$

**Table 1:** Summary of faecal sludge quantities in GAMA and Kasoa.

Faecal sludge quantification		L/cap/yr
Q_1_	Excreta generated	604
Q_2_	FS generated	4,137
Q_3_	FS collected, not delivered	0 *
Q_4_	FS collected and treated	
	Only Lavender Hill FTP	129^a^, 148^b^
	All treatment plants	213^a^, 244^b^

Note: The estimates were based on ^a^ FS collected in GAMA only; ^b^ FS collected from both GAMA and Kasoa. The Q_4_ estimates were based on projected population for 2018 based on figures inferred from [38] and [37] for Greater Accra and Kasoa respectively; * was based on FS collected by suction trucks only.

*P*_*(served)*_ is the population served by septic tanks and pit latrines; the urine generation rate (Q_(urine)_) is 1.42 L/cap/day [6]; and the estimated faecal production rate (Q_(faeces)_) is 0.236 L/cap/day for low-income countries [19].

(2) $$F\text{S ​}production\text{ ​ ​}\left(Q_{2}\right)=Q_{1}+Total\text{ ​}containment\text{ ​}\inf low_{\left(septic\text{ ​}t\text{ank}+pit\text{ ​}latrine\right)}$$

(3) $$Containment\text{ ​}\inf low=P_{\left(served\right)}\times C_{w}\times \varphi_{\text{c}}\text{ ​}+{\text{W}}_{\text{g}}\text{ ​into ​containment}$$

(4) $${\text{W}}_{\text{g}}\text{ ​into ​ ​containment}=P_{\left(served\right)}\times {\text{W}}_{\text{g}}r\times \rho_{w}\times \omega_{\text{g}}\text{ ​}$$

*C*_*w*_ is the average water consumption in GAMA estimated at 58.6 L/cap/day from supplementary data provided by [13]; *ϕ*_*c*_ = 0.05 is the proportion of *C*_*w*_ that enters a the contain system (for both septic tanks or VIPs) [13]; *W*_*g*_ is the waste generation; *W*_*g*_*r* = 0.74 kg/cap/day, the solid waste generation rate in Accra [43]; *ρ*_*w*_ is the density of solid waste assumed to be 1000 kg/m^3^ [19]; and *ω*_*g*_ = 0.01 is the proportion of *W*_*g*_ that enters the containment systems [24, 25, 26, 27, 28, 29, 30, 31, 32].

Eq. (5) was used to determine the FS collected and treated in GAMA (Q_4_), at all treatment plants and the Lavender Hill FTP. Also, the volume of FS received at a treatment facility was computed by Eq. (6) [44].

(5) $$FS\text{ ​}collected,\text{ ​}treated\text{ ​}\left(Q_{4}\right)=\frac{{\text{N}}_{\text{d}}\text{ ​}\times {\text{V}}_{T}\text{ ​(}L\text{)}}{P_{\left(served\right)}\times n\text{ ​}\left(yr\right)}$$

(6) $$FS{\text{ ​}}_{volume}\text{ ​ ​}={\text{N}}_{\text{d}}\text{ ​}\times {\text{V}}_{T}\text{ ​(}m^{3}\text{)}$$

*N*_*d*_ is the number of discharge events; *V*_*T*_ is the estimated average volume of FS per discharge event, and *n* is the number of years considered.

#### 2.3.2 Spatial analysis of faecal sludge collection

Before the spatial analysis, we grouped all the valid FS sources (neighbourhoods) recorded under their respective MMDAs, guided by a shapefile which defined the territorial boundaries and the census data. Next, Eq. (6) was used to compute the volume of FS discharged by a neighbourhood or district. The daily FS discharge was obtained by dividing the volume by the number of days in the study period (178 days).

To be able to compare the rate of emptying of FS containment systems among the administrative regions or neighbourhoods, the discharge frequency has to be normalised to factors such as the socio-economic levels, population density and the type of containment technologies adopted in the areas. Such normalisation could indicate the relative levels of service delivery by cesspit tanker operators in various areas [20].

Unlike the other administrative areas, almost all the FS collected in the Accra Metropolis are discharged at the Lavender Hill FTP. Thus, we used the Accra Metropolis as a case study. In the Accra metropolis, population density negatively correlates with income levels. On the extremes of a 5-scale categorisation by CHF International, the areas of population density below 5,000 cap/km^2^ earn above 10 USD per day and while those above 30,000 cap/km^2^ have less than 10 USD per day [45]. Hence, the level of vacuum tanker service delivery could be related to the discharge frequency normalised to the population density of the population. The normalised discharge frequency (*ND*_*f*_), relative to the population density (*ρ*_*d*_) of a neighbourhood was calculated by Eq. (7) [20].

(7) $$ND_{f}\left(km^{2}/cap\right)=\frac{{\text{N}}_{\text{d}}}{\rho_{\text{d}}\text{ ​}\left(cap/km^{2}\right)}\times 1000$$

## 3 Results and discussion

### 3.1 Truck ownership and OSS emptying practices

Records from the Lavender Hill FTP showed that 148 vacuum trucks provide the emptying and haulage services in GAMA and its environs. The number found in this study was higher than that reported in 2017 (about 120 trucks) [14]. Unlike the figure by Mansour and Esseku [14], which was based on only trucks registered with the AMA through two service operator associations, the number in this study covers all the trucks that discharge at the Lavender Hill FTP, including those outside the AMA and those owned by the quasi-state institutions. The service coverage of vacuum tanker operators in GAMA is 0.11 trucks/km^2^, which implies that one vacuum truck serves every 10 km^2^ area or every 25,000 people in GAMA.

Fifty-two (52) truck drivers, representing 86% of the targeted sample size and 35% of the number of truck drivers deduced from this study, responded to the survey questionnaire. The majority (98%) of the trucks sampled were privately owned, with the remainder being state-owned. Though parastatal institutions like SSNIT and the security services (police, army, prison, and so forth) have vacuum trucks [17], none of those trucks was encountered at the time of the survey. However, the records available at the plant confirmed the results of our survey, as only 4 out of the 148 trucks belonged to a state institution. Similar observations regarding the dominance of the private sector in providing emptying and haulage services have been made in other African countries like Kenya and Uganda [20, 46]. Over the years, FS collection and transport in Ghana was done by the waste management departments of the districts, alongside the private operators and state-related institutions [47]. However, the allocation of resources for sanitation management by the government has been very meagre [48]. The lack of funds to manage and maintain the state-owned FSM logistics and infrastructure is a major reason why the sector has been dominated by private operators in Accra [17].

On the emptying practices, a total of 89% ((always (35%), in most cases (54%)) of the drivers indicated that their tankers get full before emptying at the plant; 10% said the trucks are mostly partially filled, and less than 2% were not sure. There are usually level gauges on the tankers that are used to determine whether the trucks are full or not. Also, some of the trucks of bigger capacities (≥15 m^3^) usually empty the containment tanks of public toilets, filling the trucks to capacity in all trips. In a trip, only one containment system is usually emptied. Sometimes, the operators may top-up their partially filled tanks by emptying another system in the same neighbourhood; but according to the drivers, this seldom happens. Hence, generally, each emptying event is from only one containment system and one locality.

The contents of the containment systems are not always entirely emptied by cesspit tankers [18]. Similarly, in this study, only 25% of the respondents said that the systems are always almost completely emptied. That said, a significant proportion of them (60%) indicated complete emptying in most cases, 5% said quite often, with the remaining indicating otherwise. According to the drivers, depending on the type and size of the containment system, a simple peep through the access holes or the use of a long rod may be used to check if the tank is empty, but this is usually impossible for large systems like the public VIPs. Also, the drivers explained that depending on the truck volume and size of the containment system, more than one trip might be required for complete emptying of the system. In a few cases, the systems may be partially emptied because the owners cannot afford the cost of more than one or two trips. Furthermore, most (60%) of the drivers add water to the containment systems during the suction process. Though water is used to clean the suction hose after suction, it is mainly added to dilute the FS in dry sanitation systems (pit latrines and VIPs) during desludging. This finding corroborates earlier reports [12, 49].

Moreover, all the truck drivers indicated that they had never discharged the collected FS into the environment since the decommissioning of the Old Lavender Hill dumping site. Depending on the location of desludging, the collected FS is sent to the Lavender Hill FTP or the other treatment plants.

### 3.2 Truck capacity

**Fig. 3:**
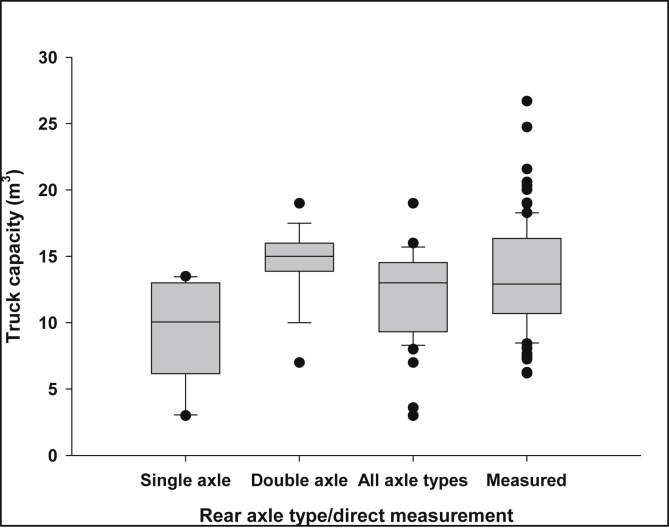
Capacities of vacuum trucks in GAMA based on the number of rear axles and direct field measurement. Black solid line in the box and black dots indicate the median and outliers, respectively.

Only 46% of the truck drivers knew the actual capacity of their trucks. The main reasons the others gave for not knowing the truck capacity were that (1) the trucks are mostly imported second-hand vehicles which come with no label or documentation on the capacity; and (2) in some cases, the tanks are locally built but without any formal design-specifications given. With that being said, those who could not tell the capacity of their trucks could relate the volume of their trucks to the rear axle type, whether single or double. The number of axles usually correlates with the load capacity of the trucks [50, 51]. For the trucks with known sizes, the capacity of both the single-axle and double-axle trucks varied, but the latter showed a wider variation, as shown in Fig. 3. The volume of the single-axle trucks ranged from 3.0 m^3^ to 13.0 m^3^ (median = 10.1 m^3^), whereas that of the double-axle trucks ranged from 7.0 m^3^ to 19.0 m^3^ (median = 15.0 m^3^). An earlier study [49] reported that the double-axle trucks used in the Madina township, in GAMA, had 20 m^3^ capacity. Using the respective median values to represent the capacities of the trucks with unknown volumes, we estimated the average truck volume at 12.0 m^3^. On the other hand, the field measurements indicated higher truck capacities: the truck volumes ranged from 6.2 m^3^ to 26.7 m^3^ (median = 12.9 m^3^). Nevertheless, in either case, the estimated truck size was higher than the average for Africa (10 m^3^) but mostly agreed with the reported range (3–25 m^3^) [52]. The average truck capacity found based on the two methods was estimated at 12.7 m^3^. Furthermore, considering the water input during the emptying process and partial filling of the tankers in some cases, we assumed arbitrarily that in a trip FS occupies at least 85% of the average truck capacity. Hence, the average FS discharged per truck was computed as 10.8 m^3^. By inference, in GAMA, the disposal of raw FS by a vacuum tanker into the environment is equivalent to 10,800 people engaging in open defecation [53].

### 3.3 Faecal sludge quantities in GAMA and its environs

The results of the different stages of FS quantification in GAMA are presented in Table 1. The estimated excreta production rate (Q_1_) and FS generation rate (Q_2_) were 604 L/cap/yr and 4,137 L/cap/yr respectively. The Q_1_ is comparable to that reported earlier for the city of Accra by Heinss et al. [15] (548 L/cap/yr) and Kampala in Uganda, also a low-income country in Africa (600 L/cap/yr) [19]; however, it is about half of that inferred from Koppelar et al. (1,285 L/cap/yr) [13] for GAMA in the year 2010. Though the Q_1_ values of GAMA and Kampala were similar, Q_2_ values of the two cities varied significantly. The FS production rate for GAMA was about one-sixth of that of Kampala (24,840 L/cap/yr) [19]. The difference primarily emanates from factors such as the distribution of the OSS types used, the assumptions made on the water consumption, pipe connections to OSS, and solid waste disposal into the systems in the two cities, during the computations.

From the results of this study's survey, no collected raw FS in GAMA is released into the environment, so Q_3_ was 0 L/cap/day. First of all, this could be attributed to the existence of several FTPs in GAMA [18]. In addition to this, the decommissioned main dumping site and the Lavender Hill FTP are only about 800 m away apart, so access to the latter does not pose any challenges. Low dumping fees serve as an incentive for discharging at the designated sites [54]. Therefore, the Q_3_ value may also be attributed to the adequately low the discharge fees collected at the treatment plants (see Section 3.6).

The FS collection and treatment rate in GAMA (Q_4_) was estimated at 213 L/cap/yr, but the FSM logistics and infrastructure (collection and treatment) handles 244 L per person every year. Heinss et al. [15] earlier reported generally higher collection rates in Accra, although the figure was for different types of sludge. The collection rates were 365 L/cap/yr, 730 L/cap/yr, and 55–73 L/cap/yr for septage, public toilet and bucket latrine sludge, and pit latrine sludge, respectively. GAMA's FS collection rate is higher than that reported for Kampala (124 L/cap/yr) [19]. The FS collection and treatment rates are remarkably lower than the generation rates because not the entire volume of the FS generated in the systems are contained for collection. Depending on the type of sanitation system used, whether wet or dry, some of FS enters the environment through soakaway pits, drain fields, drainage systems or the soil [10, 13]. However, the amount that seeps into the environment is more substantial in the case of the wet systems.

Table 2 shows the total number of FS discharge events and their equivalent estimated volumes at the six treatment plants in GAMA in the year 2018. A total of 1,006,263 m^3^ (2,757 m^3^/day) of FS was collected and treated. The Lavender Hill FTP received the majority (60.1%) of the FS collected in the study area, followed by the Teshie-Nungua WSP (22.1%), Slamson Ghana FTP (11.7%), Kotoku FTP (5.3%), Safi Sana WTEP (0.5%), and Jekora FCP (0.3%), respectively. Apart from the Teshie-Nungua WSP, which is managed by the Tema Metropolitan Assembly, all the other treatment facilities are owned and managed by private operators, though there are public-private-partnerships with the government or community involvement in some cases. Therefore, the results indicate that private companies received 77.9% of the total quantity of FS that was collected and treated the year 2018; and this further establishes that the private sector in Ghana is a major player in the management of FS in GAMA.

**Table 2:** The quantity of collected faecal sludge in GAMA and Kasoa delivered to treatment plants in 2018.

Plant	No. of discharge events	FS volume (m^3^)	% of FS volume
Lavender Hill FTP	56,203	605,057	60.1
Kotoku FTP	4,939	53,171	5.3
Jekora FCP	242	2,605	0.3
Safi Sana WTEP	474 ^*a*^	5,103 ^*a*^	0.5
Slamson Ghana FTP	10,950	117,884	11.7
Teshie-Nungua WSP	20,662 ^*b*^	222,443 ^*b*^	22.1
Total	93,470	1,006,263	100

Note: The number of discharge events were extracted from plant records spanning January to December 2018; the estimated FS volumes were based on the average FS volume per discharge (10.8 m^3^); ^*a*^ was inferred from the yearly tonnage of FS used [55], taking the density of FS from VIPs as 1001 kg/m^3^[56]; ^*b*^ was annualised based on a one-week site survey by [57] in 2018.

### 3.4 Spatial distribution of collected faecal sludge in GAMA and its environs

**Fig. 4:**
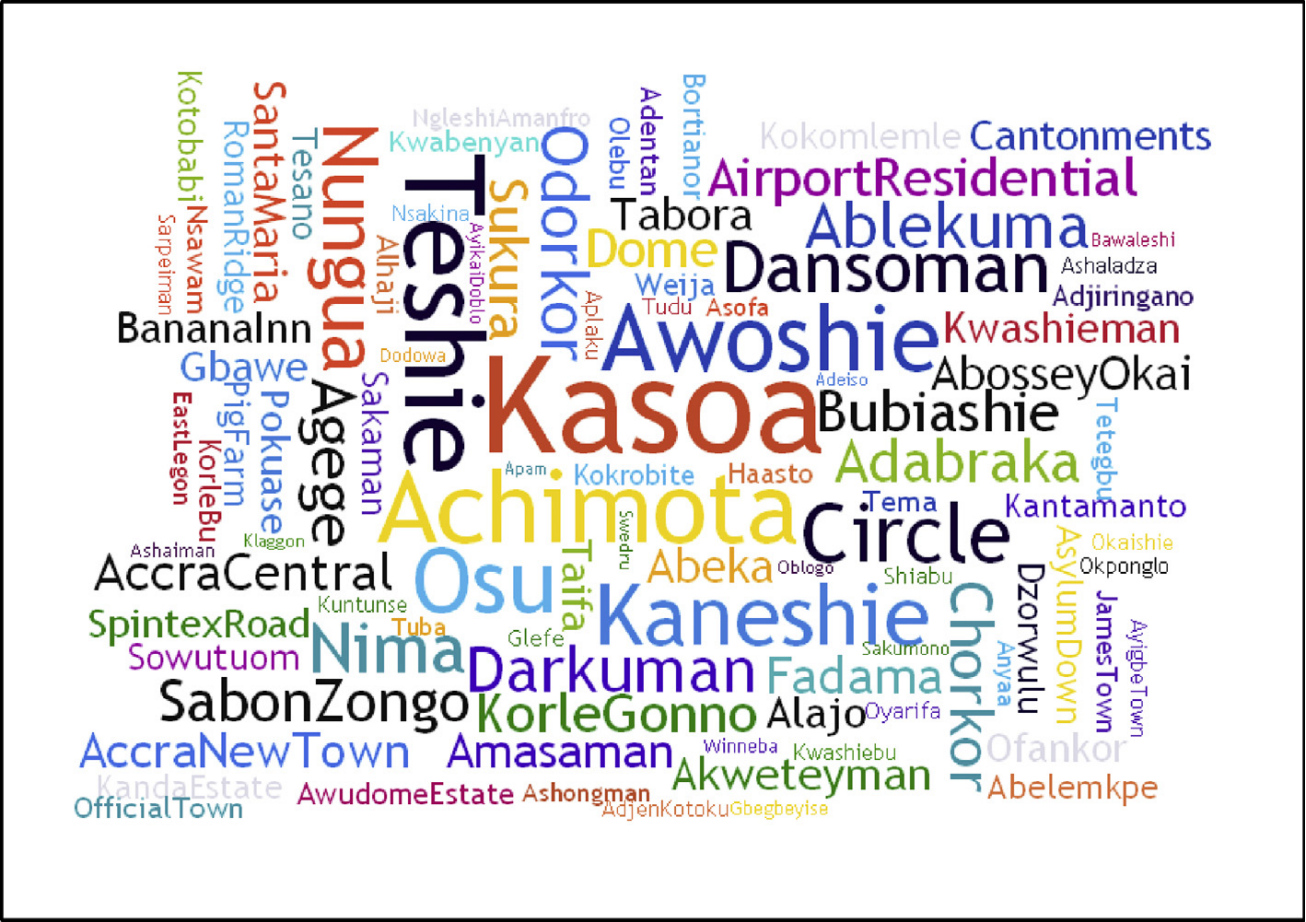
Word cloud showing the relative frequencies of discharge of faecal sludge from neighbourhoods in GAMA and its environs at the Lavender Hill FTP. Neighbourhoods with two or three names are shown as one word with a capitalisation of the first letters of the names.

This analysis was based on FS discharged at the Lavender Hill FTP. A total of 28,088 FS discharge events were collated over a 178-day study period (from 4^th^ January to 30^th^ June 2018), which equates to an average of 158 events per day and 1,697 m^3^ of FS per day. This is approximately 85% of the Lavender Hill FTP's total capacity. The volume of FS treated by the facility is more than twice the amount reported as being discharged into the Atlantic Ocean daily [58]. Koppellar et al. [13], however, estimated that about 1,300 m^3^ of FS was released into the sea daily in 2010. After screening the data, it was found that 99.2% of the data collected were eligible for the neighbourhood-sensitive analyses. 119 popular suburbs were identified as the FS sources within the study area. Fig. 4 shows a word cloud illustrating the frequency of discharge events for the various popular neighbourhoods. The top ten localities with the highest number of discharge events, in descending order, were Kasoa, Teshie, Achimota, Awoshie, Osu, Kaneshie, Kwame Nkrumah Circle area, Nungua, Agbogbloshie and Dansoman. Though parts of Osu and Dansoman are connected to the Accra CBD sewer network, they contributed to 3.65% (about 61 m^3^/day) and 2.33% (39 m^3^/day) of the total FS discharged respectively. It is also worthy to note that FS was received from the Central region (Buduburam, Winneba, Senya Breku and Swedru) as well as the Eastern Region (Nsawam and Adeiso) intermittently.

**Fig. 5:**
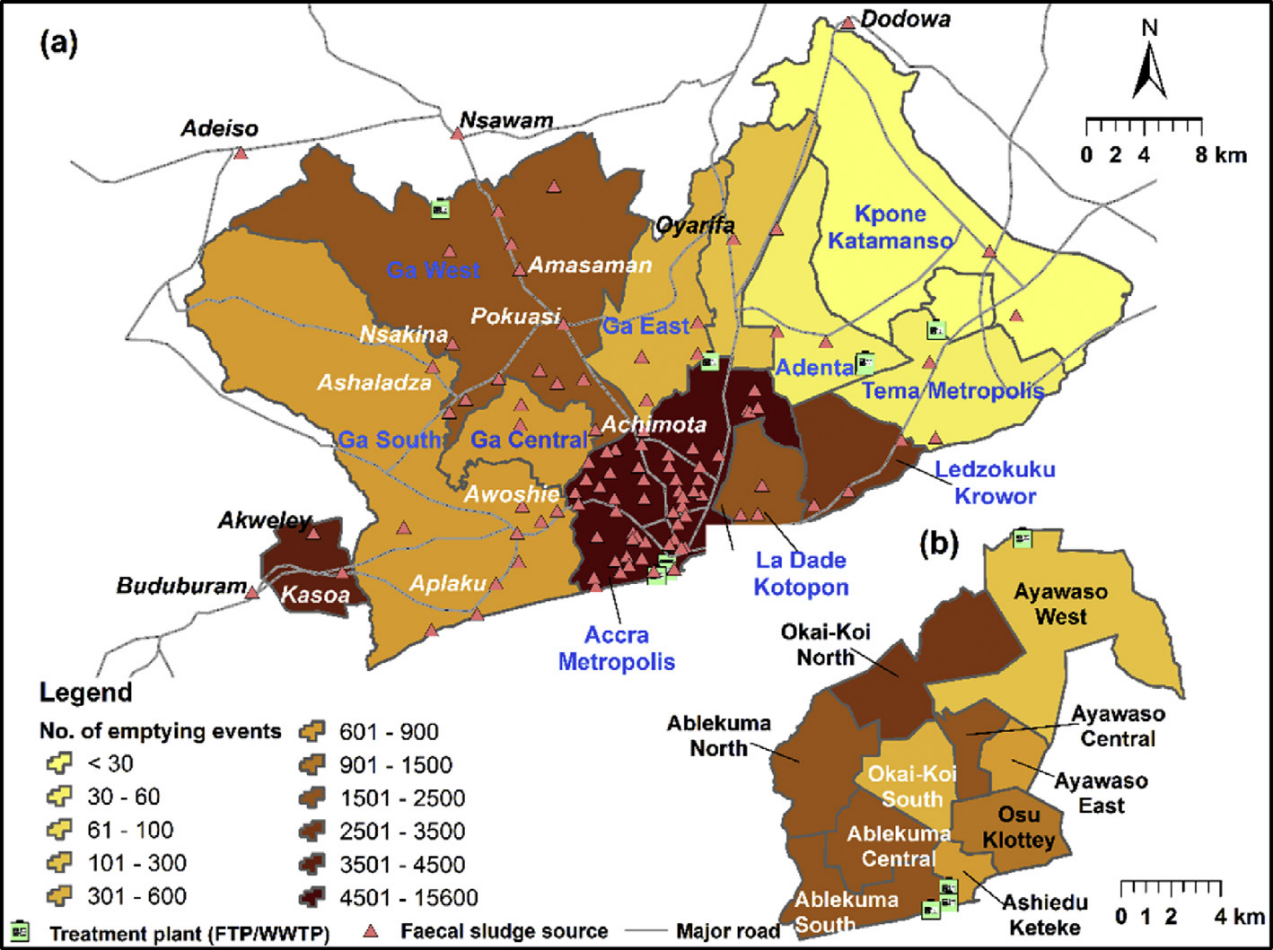
Spatial distribution of the faecal sludge sources and emptying frequency based on FS discharge at the Lavender Hill FTP. (a) Administrative areas of GAMA and Kasoa and (b) Sub-metropolises of the Accra Metropolis.

Fig. 5 shows the sources of FS and the number of FS discharge events (or emptying events of OSS) for the various MMDAs in GAMA and Kasoa. The majority (61) of these being found in the Accra Metropolis. As presented in Table 3, the Accra Metropolis recorded the highest number of emptying events, accounting for 50.56% (863 m^3^/day) of the total events. The resulting contributing regions are as follows: Ledzokuku-Krowor (11.88%), La Dade Kotopon (6.77%), Ga West (6.50%), Ga South (2.78%), Ga Central (2.68%) and the other MMDAs. Within the Accra Metropolis, the Okai-Koi North sub-metropolitan district contributed the highest (10.92%) of the discharge events. Although the Tema township is completely connected through sewers [48, 59], only 40% of the excreta generated in the Tema Metropolis enters the sewer lines which terminate at aerated lagoons [13]. The FS from OSS in the municipality accounted for 0.11% (2 m^3^/day) of is discharged daily at the Lavender Hill FTP. It is worth mentioning that the aerated lagoons (20,000 m^3^/day), which receive the wastewater from the sewer lines in Tema, are currently non-functional. This contributes to the sewage outfall into the Atlantic Ocean [8, 13, 59]. A large portion of the FS generated in Tema is transported to the Teshie-Nungua WSP, the Jekora FCP or the Safi Sana WTEP because of the convenience of their proximity. From Table 2, these three plants received an estimated average of 630.6 m^3^ of FS per day in 2018.

**Table 3:** Percentage (%) frequency of FS discharge, the volume of FS collected, and the per capita FS collection rates of the districts in GAMA, and Kasoa based on discharge events at the Lavender Hill FTP.

Metropolitan/Municipal/District	Population density∗ (cap/km^2^)	% of total emptying events	Volume of FS collected (m^3^/day)	Annualised FS collection rate (L/cap/yr)
Accra Metropolis	13,235.18	50.56	863	189
Ablekuma North^a^	12,823.74	8.78	150	278
Ablekuma South^a^	14,629.60	6.55	112	191
Ablekuma Central^a^	23,662.20	6.78	116	157
Ashiedu Keteke^a^	27,620.45	3.05	52	162
Ayawaso East^a^	33,363.27	2.43	42	83
Ayawaso West^a^	1,786.37	0.95	16	84
Ayawaso Central^a^	23,614.07	7.91	135	346
Okai Koi North^a^	11,123.23	10.92	186	298
Okai Koi South^a^	10,346.65	1.40	24	72
Osu Klottey^a^	10,618.77	4.96	85	254
Adentan	1,003.50	0.19	3	20
Ashaiman	10,297.43	0.01	0.2	0.5
Ga Central	2,392.38	2.68	46	154
Ga East	3,031.37	1.53	26	70
Ga West	877.04	6.50	111	198
Ga South	1,420.68	2.78	47	49
La Dade Kotopon	5,093.35	5.75	98	205
Ledzokuku-Krowor	4,790.99	11.88	203	354
La-Nkwantanang-Madina	1,578.94	0.40	7	24
Kpone Katamanso	474.2	0.004	0	0.3
Tema Metropolis	3,334.28	0.11	2	2
Kasoa b	1,989.22	14.42	24	1,241

a Sub-metropolitan district of the Accra Metropolis.

b Community located outside GAMA, in the Central region. Population data obtained from [33].

∗ Population refers to the number of residents who use WC, pit latrines and public toilets (WC and public toilets may be associated with pit latrines or septic tanks) extracted from the 2010 population data.

It was also observed that although the Ashaiman Municipality is densely populated, it contributed to only 0.01% (0.24 m^3^/day) of the FS recorded at the Lavender Hill FTP. Meanwhile, 95.1% of the households in the municipality use unsewered private or public toilets, including ventilated improved pits, water closets and pit latrines [25]. According to the operators of the Safi Sana WTEP, the FS used by the facility are mostly from public toilets in the municipality [55]. This signifies that both the Safi Sana facility and the Teshie-Nungua ponds receive the majority of the FS from the area.

Table 3 presents the estimates of the rate of FS collection per person for the districts and sub-districts in GAMA and surrounding areas from this study. Based on the records of the discharge events at the Lavender Hill FTP, the Ledzokuku-Krowor Manucipality had the highest per capita FS collection rate (354 L/cap/day), followed by the La Dade Kotopong (205 L/cap/day), Ga West Municipality (198 L/cap/day), Accra Metropolis (189 L/cap/day) and the Ga Central Municipality (154 L/cap/day). The collection rates for the other districts were ≤70 L/cap/day. The collection rates of the sub-districts of the Accra Metropolis ranged from 72–346 L/cap/day with the Okai-Koi South and the Ayawaso Central sub-metros having the lowest and highest rates respectively.

It is worthy to note that the Kasoa area disposed of 14.4% (246 m^3^/day) of the total volume of FS received at the Lavender Hill FTP, with a significantly high collection rate of 1,241 L/cap/day. We attribute the vast difference observed to the relatively high population growth rate (>3.3%) [37] in the Kasoa area as compared to the 2.5% of the Greater Accra Region [38], using the 2010 population census data as a reference.

The Kpone Katamanso District recorded only one discharge event during the study period. However, 53.1% of the populace use toilet facilities, either public or at the household level, which are connected to on-site sanitation containment systems [26]. The majority of FS is transported to either the Teshie Nungua or the Safi Sana facility. Similarly, the amount of FS from the La-Nkwantanang-Madina Municipality disposed of at the Lavender Hill FTP is relatively low (0.35%). This observation is because, as noted by Antwi-Agyei and others [60], the cesspit emptiers in the Madina area dispose of collected FS at the Teshie-Nunga ponds. Noticeably, 14.85% of the emptying events, representing 261 m^3^ of the daily FS discharge at the Lavender Hill FTP is from Kasoa, in the Central region of Ghana. This finding supports the recommendation by Schoebitz et al. [20] that administrative boundaries should not limit the planning of FS infrastructure and emptying service delivery.

Furthermore, we observed that the sources of FS both in and outside GAMA are close to or located along the major road network (see Fig. 5). It, therefore, implies that access to good road networks plays a critical role in the FS management. While it reduces the ease of the delivery of cesspit emptying services to an area, it decreases the travel time, the cost of maintenance of the vacuum trucks and consequently the cost of transportation. As a result, more households will be able to afford the FS emptying services, as compared to locations with poor road access.

### 3.5 Influence of population density and income levels on FS collection service coverage

Factors such as population density, income levels and the type of sanitation systems used in an area may be pointers of the levels of service provision by FS truck operators [20]. The number of people in a unit area (or population density) significantly determines the amount of faecal generation over a specified period. Consequently, this affects the rate of emptying the containment technology, especially if OSS is the dominant sanitation system.

**Fig. 6:**
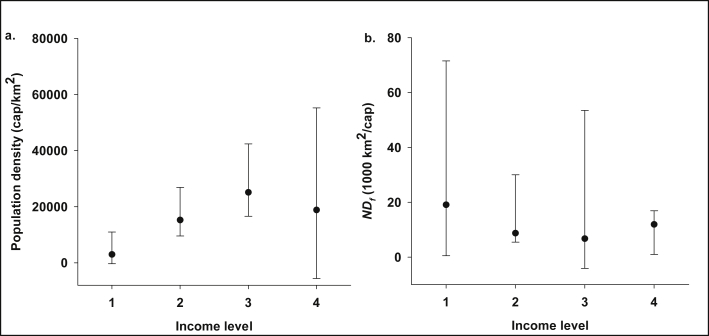
Relationship between income level and (a) population density, (b) normalised FS discharge frequency (ND_*f*_). Income levels are based on [34] and decrease with increasing number. Plots represent median and Q5,95 values.

From the results, densely populated areas in the Accra Metropolis generally have lower income levels and vice versa (Fig. 6a). Also, upper echelon neighbourhoods generally had higher normalised discharge frequencies (Fig. 6b) indicating higher service delivery. However, from Fig. 6, the suburbs with the lowest income levels were not necessarily the most congested. In densely populated communities, especially slums, service delivery by vacuum tankers may not only be limited by the low-income level of the inhabitants but also poor accessibility to the containment systems by the trucks [14, 46]; hence, manual emptying may dominate. Nonetheless, the results of this study seem to suggest otherwise: the suburbs of the lowest income had slightly higher normalised discharge frequencies as compared to those in the middle-income level. However, the difference between the normalised discharge frequencies of the two income groups was not statistically significant. We find this outcome to be quite perplexing and, therefore, provide possible explanations. Firstly, public toilet facilities dominate the neighbourhoods of the lowest income levels (such as Agbogbloshie, Gbegbeyesi, Chokor and Accra Central) rather than household toilets [61, 62] due to the lack of available space [62]. The FS discharge records also corroborate this, as the proportion of the discharge events from public toilet facilities from Chorkor and Agbogbloshie were 77.3% and 90.4%, respectively. The high patronage of the communal facilities implies that the containments fill up faster, increasing the rate of emptying, which also comes at a cost. However, in this case, the cost of FS emptying and transportation is not borne by individual households but the community, through fees charged when they access the facilities. That notwithstanding, the availability of the communal toilets does not necessarily indicate proper sanitation, as the environment of the facilities is often unhygienic [62]. Secondly, the poor suburbs are relatively closer to the Lavender Hill FTP (5.5 ± 1.2 km away) as compared to the average haulage distance in the metropolis (8.7 km). Specifically, Agbogbloshie and Chorkor, which accounted for about 85% of the FS collected in the most impoverished neighbourhoods, are only 4.5 km and 4.9 km respectively away from the plant. Hence, the FS collection charges would be quite affordable for such communities. Thirdly, the results tend to corroborate the findings of previous studies [63, 64] that the worst slums in Accra are not necessarily the most vulnerable in all aspects.

### 3.6 Optimisation of faecal sludge collection and transport

Sustainable transportation entails any means of transport that has economic and social benefits, as well as poses minimal to no impact on the environment [65]. Some key indicators of sustainable transportation are the reduction in air pollution, greenhouse gas emissions and transportation cost [65]. Longer haulage distances imply increased air pollution, GHG emissions and transportation cost. From our search, currently, no threshold has been set by any international body as the maximum sustainable haulage distance for FS transport. However, Gill et al. [22] recommends 25 km as the maximum sustainable distance, though this was based on conditions in Ireland, which may be different from developing countries. Tayler [44] recommends that in developing countries, the haulage distance should not exceed 15 km. UN-HABITAT [66] suggests 20 km as the maximum travel distance for the collection and transport of municipal solid waste (MSW) in developing countries; and in many of such countries, FS dumping sites or treatment plants may be sited on the same premises [44]. So, we could infer that the recommended maximum distance for MSW haulage is comparable to that of FS. Thus, generally, the longest sustainable distance ranges from 15 to 25 km.

With the aid of GIS tools, FS collection and haulage can be optimised to help increase the sustainability of FSM in an area. The tools help in (1) identifying areas of service coverage, (2) reducing the cost of transporting of faecal sludge [20], and (3) siting new faecal treatment facilities for optimal socio-economic and environmental benefits.

#### 3.6.1 Minimising the travel distance

The results of the road network analysis, presented in Table 4, indicate that in GAMA, the vacuum tankers travel 0.7–42.5 km (mean = 14.2 ± 9.2 km) using 6.7–68.7 min (average = 32.7 ± 15.7 min) to discharge at the Lavender Hill FTP. We found that the FS sources located outside GAMA are about 35 km–88 km away from Lavender Hill FTP. FS from about 39% and 29% of the neighbourhoods was hauled over 15 km and 45 minutes respectively, as recommended by Tayler [44] for low- and middle-income countries. Also, 20% of the suburbs, which included all the FS sources outside GAMA, fell outside the sustainable range for FS haulage. These neighbourhoods accounted for 269 m^3^ of the daily FS discharge at the facility. Over the study period, the farthest FS source was Swedru in the Central region of Ghana, which is about 88 km away from the Lavender Hill FTP. Swedru is also connected to the main road network. Therefore, as pointed out in Section 3.1, accessibility to good roads increases the coverage by the FS emptying service providers in the area.

**Table 4:** Average distance and minimum time of FS transport from the districts to the Lavender Hill FTP.

	Metropolis/Municipal/District	Average distance travelled (km)	Minimum transportation time (min)
Greater Accra Metropolitan Area (GAMA)	Accra Metropolis	8.71	23.12
	Ablekuma North^a^	12.26	32.40
	Ablekuma South^a^	5.53	16.54
	Ablekuma Central^a^	5.99	15.10
	Ashiedu Keteke^a^	4.07	14.98
	Ayawaso East^a^	10.10	28.35
	Ayawaso West^a^	15.65	35.12
	Ayawaso Central^a^	9.30	24.07
	Okai Koi North^a^	13.22	34.71
	Osu Klottey^a^	6.63	19.43
	Adentan Municipal	22.95	49.50
	Ledzokuku-Krowor	20.45	42.35
	La-Nkwantanang-Madina	26.28	55.43
	La Dade Kotopon	10.93	25.90
	Ga Central	20.16	46.88
	Ga East	21.30	49.56
	Ga West	26.22	50.28
	Ga South	18.64	40.16
	Kpone Katamanso b	39.42	70.00
	Tema Metropolis b	42.50	68.70
	Ashaiman b	34.27	59.00
Central region	Kasoa b	31.80	56.00
	Buduburam b	34.70	62.00
	Swedru b	87.5	144.30
	Winneba b	62.80	94.00
	Senya Breku b	54.90	97.00
Eastern region	Nsawam b	42.70	70.00
	Adieso b	57.90	74.50

a Sub metropolitan district of the Accra Metropolis.

b Neighbourhood or MMDA outside the 25 km sustainable distance.

The number of discharge events from the La-Nkwantanang-Madina Municipality is equivalent to only about 7 m^3^ per day, but the suction trucks travel an average of about 26.3 km to the Lavender Hill FTP. Similarly, the Adentan municipality also discharges only about 3 m^3^ per day. Some of the FS collected from these two districts are disposed of at either the Jekora FCP or the Teshie-Nungua WSP [49]. However, according to the operators of the Jekora FCP, the vacuum trucks are only called to discharge at the facility when FS is needed for composting. Thus, the plant received only 242 trucks in the year 2018 (about five trucks per week). Considering the relatively small volumes of FS transported to the Lavender Hill FTP and the proximity of these two municipalities to the Legon WSP, which currently operates less than 20% of its capacity, the ponds can receive and treat faecal sludge from those municipalities. However, since the FS is of relatively high strength, it is essential to conduct preliminary studies on the effect of the desludged material on the operational efficiency of the Legon ponds. Typical chemical oxygen demand (COD) levels of FS range from 1,200 to 50,000 mg/L, with the total solids (TS) levels from 12,000 to 52,500 mg/L [18]. However, the COD values of FS received at the Lavender Hill facility averages to around 18,000 mg/L. Moreover, adequate primary screening will be needed because the faecal sludge generated in Accra has significantly high levels of municipal solid waste and grit [67].

The Kotoku FTP is located in the Ga West municipality and is, therefore, likely to receive FS in this area. For the Ga West district and its environs, the Kotoku FTP is much closer and is more favourable to vehicular traffic, apart from the facility currently operating at only about 20% of its treatment capacity. Conversely, on the average, 15.5 m^3^ of FS is transported over 25 km (maximum sustainable distance) from the Ga West to the Lavender Hill FTP every day. Occasionally, the cesspit trucks bypass the Kotoku FTP and travel an average of 50 km from the Eastern region to the Lavender Hill FTP. Since the travel distance and time affect the haulage cost [16], this study also investigated why some of the vacuum truck operators opt for the Lavender Hill FTP over the Kotoku FTP, as a way of optimising FSM in the municipality and GAMA as a whole. The responses from the survey showed that though 98% of truck drivers were aware of the existence of the Kotoku FTP, 58% of them had never discharged their loads (FS) there before. The majority (62%) of the remaining 40% who have discharged at the Kotoku FTP, preferred the Lavender Hill FTP to the Kotoku FTP, assuming the distance from their FS collection point to the two plants is the same. The reasons given for this included: (1) significant service delays at the Kotoku plant; (2) frequent truck breakdowns, especially for the single-axle trucks, due to the several speed ramps on the road to Kotoku FTP; and (3) the relatively organised service delivery at the Lavender Hill FTP.

Moreover, minimising the FS transport distance helps to reduce the haulage cost, making the services more affordable to households and economically attractive to the vacuum truck operators. Various reports indicate that vacuum tanker operators may dispose of FS at inappropriate sites if the distance to the FTP is too long [5, 16, 68, 69, 70].

From this study, Kasoa is a business hub for vacuum tanker operators and promises to be the same in the future, considering the population growth rate and the rate of development in the area. However, the cost burden on the inhabitants in managing FS will also continue to increase due to the long distance (31.8 km) and the vehicular traffic situation on its main route to the Lavender Hill FTP. If no intervention is implemented with time, the inhabitants may find other ways of disposing of their waste, which may be detrimental to human health and the environment. It is, therefore, recommended that a local FS treatment system be used for Kasoa and its neighbouring communities.

Similarly, though the Ledzokuku-Krowor municipality has waste stabilisation ponds in Teshie-Nungua, the ponds are unable to handle the FS load from the growing population in the area, hence affecting the treatment efficiency of the plant [9]. This resulted in the observed 161 m^3^ of FS from Teshie being discharged daily at the Lavender Hill FTP. To considerably help optimise FS management in the municipality, by reducing the haulage distance and related costs, an increase in the capacity of the plant or new technologies needs to be added.

#### 3.6.2 Regulating the cost of faecal sludge collection and haulage

From the results in Section 3.1, private operators dominate the FS collection and transport market in GAMA. According to the 2010 National Environmental Sanitation Policy [71], private entities can undertake “*the provision and management of septage tankers, on a fully commercial basis subject to the licensing and the setting of maximum tariffs by the Assemblies.*” However, to date, though tariffs have been set for the hiring of trucks owned by the MMDAs and are revised annually in the Imposition of Rates and Fee-fixing Resolution documents of the assemblies, there is no tariff regime available for the private operators.

At the time of the study, the cesspit emptying operators in GAMA charged GHC 150–600 (30–125 USD) per trip. According to the truck drivers, the cost is chiefly determined by the travel distance, the truck capacity, the nature of the roads to the FS source, and the travel time which partly depends on the level of traffic congestion of the route. Boot and Scot [12] noted that fuel consumption depends on the haulage distance and accounts for one-third of the total cost of a trip in Accra. However, in general, the cost of FS C&H in GAMA is relatively high as compared to other African and South Asian countries, which also fall in the low-to middle-income category, though this comparison is not strictly based on the same conditions (e.g. travel distance, truck capacity, etc.) in the cities. The maximum FS C&H cost in those cities is usually about USD 60. For example, in Dakar, Senegal, it costs households 50 USD to desludge 10 m^3^ of FS [72], while in the peri-urban areas of Addis Ababa, Ethiopia, the cost ranges between USD 9.3 and USD 36.0 [46]; in Kisumu, Kenya, mechanical emptying costs averagely USD 52 [52]. Also, in urban India, house owners pay USD 25–30 per trip for FS collection and haulage [70].

Meanwhile, in GAMA, the operators pay a discharge fee ranging from GHC 15–30 (USD 3.1–6.2) per trip depending on the capacity of the truck; their counterparts in Kampala pay almost the same discharge fee (USD 2.0–5.6) per trip. However, the cost of haulage is relatively low in Kampala because the maximum charge of haulage per trip is USD 45 [20], [54], though higher costs (about USD 24–60) have also been reported in the informal settlements of Kampala within a 5 km distance [73]. The relatively high cost of FS collection and transport can partially be attributed to the inexistence of a structured haulage cost regime which allows the service providers to charge arbitrarily. In the absence of tariffs set by the MMDAs, the leaders of the associations of vacuum tanker operators could somewhat regulate their members' price charges for an area, as it is the case of other transport unions such as the Ghana Private Road Transport Union (GPRTU), the Progressive Transport Owners Association (PROTOA) and so on, for commercial passenger vehicles. However, similar to the case of Kampala [73], the heads of the associations are also truck owners, and hence are unable to regulate the prices. Although the associations of the vacuum tanker operators are under the Environmental Services Providers Association (ESPA), which somewhat regulates the activities of its members, the service charges are determined by the individual service providers. Therefore, as the demand increases, the service charges may be increased arbitrarily. Consequently, households that cannot afford the services of the cesspit emptiers may resort to inappropriate methods of disposal, which could result in environmental pollution and human health concerns [68]. Chinedu et al. [23] noted that reasonable and affordable price and tariff structures are crucial determinants of achieving sustainable urban sanitation in Africa. Thus, the MMDAs in GAMA need to exercise their mandate according to the National Sanitation Policy [71] by setting reasonable cost limits for their jurisdictions. The results of the road network analysis (see Table 4) provides fundamental data to estimate the cost of FS collection and haulage for the MMDAs. However, we recommend broad consultations with all stakeholders, including the vacuum tanker operators during the tariff setting process.

Aside from regulating the cost of FS collection and transport, experience from Kampala shows that services rendered by government trucks are usually cheaper [54]. Though this is replicable in Accra, the low expenditure by the government of Ghana to ensure sustainable operation and maintenance is a challenge [17]. Therefore, we recommend that the government commits to the provision of vacuum tankers with adequate funding and personnel to all the MMDAs to ensure the delivery of subsidised FS collection and haulage services. Besides, poorer neighbourhoods must be given a priority in the delivery of such services. This approach will help reduce the likelihood of households engaging illegal emptiers who discharge FS into the environment [5, 16, 68, 69, 70].

### 3.7 Limitations of the study and recommendations for future research

Although current (the year 2018) data was used on the collected FS in this study, the population figures used were from the 2010 population and housing census, because though projected population data were available details required for this study were missing. Thus, the per capita collection rates computed in this paper may be overestimated. Also, the spatial analysis was based on only discharge events recorded at the Lavender Hill FTP, which is a sink for about 60% of the FS collected in GAMA. As a result, the estimate (collected FS volumes and per capita collection rates) reported for some of the districts (especially the La-Madina Nkwantanang, Ga West, Ashaiman and Tema municipalities), which have other FS disposal options, may not be truly representative. It is also important to note that the Q_3_ (FS collected but not delivered for treatment) reported in this study was based on the FS collected by vacuum tankers only. Nonetheless, manual emptiers may be engaged in low-income areas or slums where households may not be able to afford mechanised emptying or accessibility of the suction trucks to the containment system is a challenge [14, 52]. In such instances, the FS removed is not transported to a treatment facility but discarded directly into the environment affecting the FS quantification values [18]. Hence, the amount of FS emptied but not collected needs to be quantified by future studies.

Moreover, the travel times presented in this study are the minimum values and may change depending on the vehicular traffic situation on the route used.

For the design of FS treatment infrastructure, data on both the accumulation rate of the containment systems and the collection rate in the catchment area are critical [19]. Therefore, it is recommended that further studies be conducted on the accumulation rates of OSS containment systems to fine-tune the planning of FS logistics and infrastructure in GAMA.

## 4 Conclusions

The study has presented the case of the Greater Accra Metropolitan Area (GAMA) and its environs, in Ghana, regarding faecal sludge (FS) collection and haulage by employing GIS tools. Based on information from the literature, we quantified the excreta and FS generated daily in the study area. Also, data were collected from the treatment plants in the study area to help quantify the amount of FS collected and sent for treatment. Moreover, records obtained from the Lavender Hill FTP on the FS discharge from the neighbourhoods were used to map the collected FS to the districts in GAMA and its environs. Also, the average travel distances and times from the neighbourhoods and districts to the Lavender Hill facility were estimated. Furthermore, by using the Accra Metropolis as a case study, we investigated the relationship between the level of income of communities and the delivery of FS collection and haulage services.

The results indicated that 604 L of excreta is generated per person in GAMA every year, with an FS production rate of 4,137 L/cap/year. Also, 213 L of FS is collected for treatment per person annually. Moreover, the private sector dominates the FS collection, haulage and treatment markets in GAMA and its neighbouring towns, possibly because of the relatively low government investment in the sector. One FS truck serves 25,000 people in GAMA. It was estimated that the average vacuum truck capacity is 12.7 m^3^, but the average volume of FS per discharge event was 10.8 m^3^. This study also shows that the Lavender Hill Faecal Treatment Plant (FTP) currently does not serve only areas in the Greater Accra Metropolitan Area, but parts of the Central and Eastern regions of Ghana as well. The facility treats an average of 1,697 m^3^ every day, which is about 85% of its design capacity. The suction trucks travelled less than 1 km–88 km to discharge at the Lavender Hill plant. About 270 m^3^ of FS disposed of at the facility is from neighbourhoods with haulage distances longer than the maximum sustainable distance (25 km). Moreover, this study has provided fundamental data on the FS haulage distances and time from each district to the Lavender Hill FTP to help in the process of rationalising the cost FS collection and haulage in GAMA by all stakeholders. Moreover, we recommend that the government makes adequate investment in FS collection and transport logistics and human resource to promote the provision of subsidised services in all the districts in GAMA. The study also revealed that Kasoa, a peri-urban community of the Greater Accra region, contributes to about 14.4% (246 m^3^/day) of the total FS discharged daily at the Lavender Hill FTP, highlighting the importance of looking beyond administrative boundaries when planning for FS management logistics and infrastructure. Finally, the income levels and population densities of localities in GAMA relate to FS the discharge frequency or the level of service delivery by the suction truck operators. However, the most impoverished communities are not the least-served, regarding FS collection and haulage.

## Declarations

### Author contribution statement

Gideon Sagoe: Conceived and designed the experiments; Performed the experiments; Analyzed and interpreted the data; Wrote the paper.

Felix Safo Danquah: Conceived and designed the experiments; Performed the experiments; Contributed reagents, materials, analysis tools or data.

Eric Simon Amofa-Sarkodie: Conceived and designed the experiments; Contributed reagents, materials, analysis tools or data.

Eugene Appiah-Effah: Analyzed and interpreted the data; Wrote the paper.

Elsie Ekumah: Contributed reagents, materials, analysis tools or data; Wrote the paper.

Emmanuel Kwaw Mensah, Kenneth Sefa Karikari: Performed the experiments; Contributed reagents, materials, analysis tools or data.

### Funding statement

This research did not receive any specific grant from funding agencies in the public, commercial, or not-for-profit sectors.

### Competing interest statement

The authors declare the following conflict of interests: the authors are staff of Sewerage Systems Ghana Ltd, a private company which operates both the Lavender Hill FTP and the Kotoku FTP, and the Kwame Nkrumah University of Science and Technology. However, there are no known conflicts of interest associated with this publication, and there was no financial support for this work that could have influenced its outcome.

### Additional information

No additional information is available for this paper.
